# Low dose oral beta-lactamase protects the gut microbiome from oral beta-lactam-mediated damage in dogs

**DOI:** 10.3934/publichealth.2019.4.477

**Published:** 2019-11-12

**Authors:** Sheila Connelly, Brian Fanelli, Nur A. Hasan, Rita R. Colwell, Michael Kaleko

**Affiliations:** 1 Synthetic Biologics, Inc., Rockville, MD, United States; 2 CosmosID, Inc., Rockville, MD, United States; 3 University of Maryland Institute for Advanced Computer Studies, College Park, MD, United States

**Keywords:** antibiotic, gut microbiome, beta-lactam, beta-lactamase

## Abstract

Antibiotics, while lifesaving, damage the gut microbiome and can precipitate proliferation of pathobionts. A strategy to preserve gut microbiome integrity is to eliminate biologically active antimicrobials excreted into the gastrointestinal tract (GI) without negatively affecting antibiotic therapeutic efficacy. Clinical proof of concept was achieved with SYN-004 (ribaxamase), a beta-lactamase enzyme formulated for oral delivery with intravenous penicillins and cephalosporins. Ribaxamase inactivated intestinal ceftriaxone, protected the gut microbiome, and significantly reduced the incidence of *Clostridioides difficile* disease. For use with oral beta-lactam antibiotics, a delayed release formulation of ribaxamase, SYN-007, was engineered for dissolution in the lower small intestine distal to the site of oral antibiotic absorption. In dogs that received oral amoxicillin, SYN-007 reduced microbiome disruption without interfering with amoxicillin systemic absorption. Here, a study to determine the lowest effective dose of SYN-007 was performed. Dogs received amoxicillin (40 mg/kg, PO, TID) +/− SYN-007 (PO, TID) at three doses, 10 mg, 3 mg, or 1 mg for five days. Serum amoxicillin levels, measured after the first and last antibiotic doses, were not significantly different +/−SYN-007 at all dose levels indicating that SYN-007 did not interfere with amoxicillin systemic absorption. Microbiome analyses demonstrated that amoxicillin significantly reduced bacteria richness and microbiome diversity resulting in altered microbiome composition. However, with all doses of SYN-007, microbiome richness and diversity were not significantly different from pretreatment and changes in microbiome composition were attenuated. These data demonstrate that effective SYN-007 doses can be reduced at least 10-fold while maintaining gut microbiome preservation. The potential to employ low SYN-007 doses to protect the gut microbiota has important implications for enhancing therapeutic outcomes for patients receiving oral beta-lactam antibiotics while simultaneously reducing cost per dose and ultimately, healthcare expenses.

## 1. Introduction

The gut microbiome, constituting the commensal microorganisms within the gastrointestinal (GI) tract and their genetic material, plays key roles in health and disease. Antibiotics can damage this diverse ecosystem, resulting in immediate alterations of microbiome functional composition and instigating potential long-term adverse effects on health [1]. To limit exposure of the gut microbiota to antimicrobials, beta-lactamases, bacterial enzymes that inactivate beta-lactam antibiotics, were harnessed as therapeutics to degrade antibiotics in the proximal intestine before disturbing the colonic microbiota. SYN-004, ribaxamase, is an enteric-coated formulation of a beta-lactamase enzyme, intended for oral administration with intravenous (IV) penicillins and cephalosporins that are excreted via bile at high levels into the upper GI tract [2]. In clinical studies, orally delivered ribaxamase degraded the beta-lactam antibiotic, ceftriaxone, in intestinal fluid [3], protected the gut microbiome [4], and significantly reduced the incidence of *Clostridioides difficile* infection (CDI) in patients receiving ceftriaxone for a lower respiratory tract infection [5],[6]. To broaden the utility of this approach to include oral as well as IV beta-lactams, a delayed release formulation of ribaxamase, SYN-007, was developed [7].

SYN-007 utilizes a dual coating approach, enteric-coated enzyme pellets within enteric-coated capsules, to target dissolution to the distal small intestine and prevent interference with oral beta-lactam systemic absorption [7]. SYN-007 was engineered to ensure that no enzyme release occurred in the upper small intestine, as the beta-lactamase would degrade the antibiotic prior to absorption. Premature release is not an issue for most GI site-directed drug delivery applications where slight leakage is accepted as long as most of the drug is delivered to the target site [8]. While several alternative SYN-007 preparations were evaluated in dogs, this dual-coated formulation was the only one that protected the gut microbiome without significantly interfering with oral amoxicillin systemic absorption [7]. However, close comparison of the amoxicillin serum pharmacokinetic (PK) curves following 16 doses of amoxicillin +/− SYN-007 (10 mg/dose) revealed, in the presence of SYN-007, a more rapid decrease in amoxicillin serum levels at later time points compared to amoxicillin alone [7],[9]. These observations suggest that trace amounts of the beta-lactamase were present in the upper small intestine, potentially from low-level premature enzyme release, which resulted in degradation of a minute portion of amoxicillin prior to its systemic absorption. As systemic antibiotic concentrations were affected minimally, reduced serum levels were measurable only when antibiotic concentrations had declined close to baseline [7],[9]. Therefore, a simple strategy to minimize the amount of beta-lactamase present in the upper small intestine and thereby optimize oral antibiotic systemic absorption is to deliver lower enzyme doses.

Clinically, ribaxamase has a broad therapeutic window, is not systemically absorbed, and is well tolerated [2],[3],[4],[6],[10]. The favorable therapeutic profile of ribaxamase allowed repeated dosing at high enzyme levels resulting in concentrations of 1,000,000 ng/mL detected in the intestinal fluid of some patients [3]. Ribaxamase function was evaluated clinically with IV ceftriaxone administered once a day [3],[6]. Thus, ribaxamase patient dosing regimens were chosen to achieve continuous high concentration bioavailability in the intestine [2] rather than attempting to refine doses based on variable gastric emptying and intestinal transit times [8]. In contrast, the delayed release formulation of ribaxamase, SYN-007, is intended to be administered concurrently with an oral beta-lactam following the antibiotic dosing regimen, typically, several times per day. Therefore, patient to patient variability in GI tract function is not expected to be problematic since SYN-007 and the antibiotic will transit together. As ribaxamase efficiently inactivates penicillins and cephalosporins [2], we hypothesize that doses can be reduced while maintaining effective gut microbiome protection.

Here, a SYN-007 dose response study was performed in dogs that received oral amoxicillin to evaluate the lowest effective SYN-007 dose that maintained gut microbiome integrity without interfering with oral antibiotic systemic absorption.

## 2 Materials and methods

### 2.1. Test article

The delayed release ribaxamase formulation, SYN-007, contained sugar pellets coated with ribaxamase and Eudragit^®^ L30-D55 (Evonik, Essen, Germany) [11] encapsulated into 2.69 mm diameter x 5.1 mm length (size 9 h) capsules that were banded and spray coated with Eudragit^®^ FS30D (Evonik) [7]. For the SYN-007 10 mg dose, each 9 h capsule contained eight enzyme layered pellets and eight filled and coated 9 h capsules were loaded into one size 0 uncoated hard capsule. The SYN-007 3 mg dose contained seven enzyme layered pellets per 9 h capsule with three filled banded and coated 9h capsules loaded into one size 0 uncoated hard capsule. The SYN-007 1 mg dose contained seven enzyme layered pellets per 9 h capsule with one filled, banded and coated 9 h capsule loaded into one size 0 uncoated hard capsule. SYN-007 was manufactured and tested by Aptuit, an Evotec Company, formerly Kuecept, Ltd.

### 2.2. Animals and test article administration

Animal studies were performed at Calvert Laboratories, Inc. (Scott Township, PA). Twenty healthy adult (7 to 8 months old) female Beagle dogs, 6.5 to 7.7 kg, were obtained from Covance Research Products (Denver, PA). Dogs were naïve and had never been exposed to antibiotics. Animals were acclimated for 30 days prior to their first fecal collection with health monitored daily. In compliance with USDA Guidelines, animals were individually accommodated and permitted to socialize except on feces collection days and on study days 1–6. Dogs were fed antibiotic-free PMI Canine Diet. Animal cohorts (n = 5) were: Amoxicillin alone, Amoxicillin + SYN-007 (10 mg), Amoxicillin + SYN-007 (3 mg), and Amoxicillin + SYN-007 (1 mg).

Animals were dosed exactly as described [7]. Amoxicillin was supplied as a powder (NDC 0781-6157-52) and resuspended in 100% Mott's Apple Juice (pH 3.0) instead of water, exactly as described [7].

Blood was collected from dogs after the first dose on day 1 and on day 6 at 0.5, 1, 2, 3, 4, 6, and 8 hours after antibiotic administration, and feces were collected on day 1 before antibiotic dosing and on day 6 following dosing exactly as described [7].

All animal procedures were performed in compliance with guidelines established by the Calvert Institutional Animal Care and Use Committee (IACUC) in compliance with the Animal Welfare Act at Calvert Laboratories, Inc. (Scott Township, PA). The Calvert IACUC approved the animal study protocol prior to animal acquisition and study initiation. Calvert Laboratories, Inc. is fully accredited by the Association for Assessment and Accreditation of Laboratory Animal Care (AALAC).

### 2.3. Amoxicillin serum measurement

Serum was analyzed for amoxicillin by Sannova Analytical, Inc., exactly as described [7]. GraphPad Prism7 was used for statistical analyses and Area under the curve calculations.

#### 2.3.1. Fecal DNA extraction, whole genome shotgun sequencing and metagenomic analyses

Fecal specimens collected prior to and following amoxicillin +/− SYN-007 administration were analyzed following total DNA isolation, library construction, quantification, and sequencing exactly as described [7]. Open source BBDuk software from BBTools (https://jgi.doe.gov/data-and-tools/) was used to perform standard read quality assessments and all samples conformed to an average read quality of Q20 (https://www.illumina.com/science/education/sequencing-quality-scores.html). Comparable read depth was achieved as reads/sample were 62,000,000 ± 11,000,000.

Bacterial identification and determination of species relative abundance was performed by directly analyzing unassembled whole genome shotgun metagenomic sequencing reads with the CosmosID, Inc. bioinformatics software package (CosmosID Inc., Rockville, MD), as described [7]. Microbiome diversity measurements, based on the metagenomics sequencing data, included bacterial richness, the number of bacterial species present in each fecal sample, alpha diversity based on the Shannon index [12], that measures species richness and species relative abundance, and beta-diversity using principal coordinate analysis, that measures how similar two samples are to each other relative to all other samples in the analysis. Stacked bar graphs were generated based on relative abundance of each microorganism (%) in each sample using the NMF R software package [13]. Statistical analyses were performed using GraphPad Prism 7.

#### 2.3.2. Data Availability

Fecal DNA metagenomics sequencing data are available in Sequence Read Archive (SRA) (https://submit.ncbi.nlm.nih.gov/subs/sra/), Accession SRP093227.

## 3. Results

### 3.1. SYN-007 did not significantly affect oral amoxicillin systemic absorption

**Figure 1.**
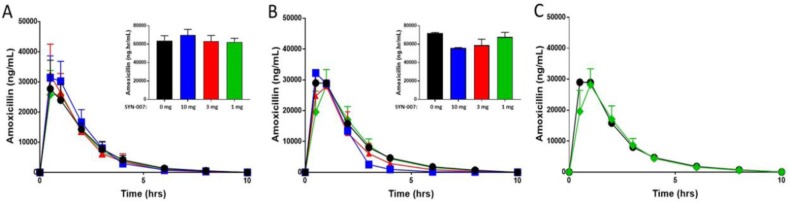
Amoxicillin serum levels. Amoxicillin was measured in dog serum collected at the indicated times. Notes: Inset bar graphs display the area under the curve (AUC) for each group. A. Amoxicillin levels after the first dose of amoxicillin (Day 1) +/− SYN-007. B. Amoxicillin levels after the last (16^th^) dose of amoxicillin (Day 6) +/− SYN-007. C. Amoxicillin levels after the 16^th^ dose of amoxicillin alone or amoxicillin + SYN-007 (1 mg). Black, Amoxicillin alone; blue: Amoxicillin + SYN-007 (10 mg); red: Amoxicillin + SYN-007 (3 mg); green: Amoxicillin + SYN-007 (1 mg). Data are displayed as mean + standard deviation (n = 5). P values were obtained by analysis of the AUC from each group for each collection day using Kruskal-Wallis non-parametric ANOVA with Dunn's multiple comparisons test (Graphpad Prism 7) comparing each group to the amoxicillin alone group for the appropriate collection day. Day 1: Amoxicillin vs. SYN-007 (10 mg), p > 0.9999; Amoxicillin vs. SYN-007 (3 mg), p > 0.9999; Amoxicillin vs. SYN-007 (1 mg), p > 0.9999. Day 6: Amoxicillin vs. SYN-007 (10 mg), p = 0.0645; Amoxicillin vs. SYN-007 (3 mg), p = 0.2845; Amoxicillin vs. SYN-007 (1 mg), p > 0.9999.

A dose response study of SYN-007, a delayed release formulation of ribaxamase, was performed in dogs. Animals received oral amoxicillin +/− SYN-007 (10 mg, 3 mg, or 1 mg) three times a day for five days with their final dose on the morning of day 6, for a total of 16 doses. Amoxicillin systemic absorption was evaluated following the initial antibiotic dose +/− SYN-007 on day 1 and the final dose on day 6. Area under the curve (AUC) for each SYN-007 cohort was compared to the AUC for the amoxicillin alone control groups for each day (Figure 1). On day 1, PK curves were similar for amoxicillin alone and all SYN-007 cohorts. Likewise, amoxicillin serum AUC in the presence of all doses of SYN-007 was nearly identical to that of amoxicillin alone (p > 0.9999; Figure 1A). By day 6, after 16 doses of antibiotic +/− SYN-007, amoxicillin PK curves remained similar for all cohorts at 30 min, 1, and 2 hours after amoxicillin ingestion. However, by 3 hours, amoxicillin levels appeared to decrease more rapidly in the two highest dose SYN-007 cohorts (10 mg and 3 mg). In contrast, with the lowest SYN-007 dose (1 mg), the amoxicillin PK curve was superimposable with that of the amoxicillin alone cohort at later time points (Figure 1C). While there was no significant difference in amoxicillin serum level AUC for any SYN-007 cohort compared to amoxicillin alone, statistical analyses demonstrated that the p-values increased as the SYN-007 dose decreased (p = 0.0645, 0.2845, and >0.9999, for SYN-007 10 mg, 3 mg, and 1 mg, respectively; Figure 1B).

### 3.2. SYN-007 attenuated microbiome damage

Gut microbiome composition was assessed following whole genome shotgun metagenomic analyses using DNA extracted from feces collected before and after amoxicillin +/− SYN-007 treatment. Bacterial species relative abundance for each sample was determined (Table 1). Species richness of all pretreatment microbiomes was 283 ± 27 which was not significantly different to species abundance of each pretreatment cohort (0.3468 ≤ p ≥ 0.9999). Following antibiotic +/− SYN-007 treatment, amoxicillin alone microbiomes showed significant decreases in bacterial abundance (p = 0.0002), while no significant decrease in species richness was detected with all doses of SYN-007 (p = 0.0519, 0.0870, and 0.4295 for SYN-007 10 mg, 3 mg, and 1 mg, respectively).

**Figure 2.**
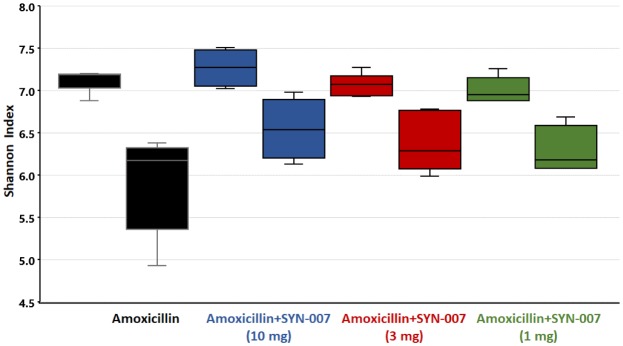
Comparison of dog fecal microbiome Shannon alpha diversity prior to and after antibiotic treatment. Notes: Fecal microbiome metagenomics data were analyzed by Shannon index and are displayed for each cohort as box plots. Cohorts were n = 5 for all but Amoxicillin + SYN-007 (10 mg), where n = 4. A pretreatment sample from one animal in this cohort (Dog 8) was considered an outlier based on Principal coordinate analysis of all pretreatment samples (Supplemental Figure 1). P values were obtained by comparing pretreatment Shannon indexes (Pre) to post-treatment Shannon indexes (Post) of each cohort using Kruskal-Wallis non-parametric ANOVA with Dunn's Multiple Comparisons test (Graphpad Prism 7). Black, Amoxicillin alone, p = 0.0063; blue, Amoxicillin + SYN-007 (10 mg) without Dog 8, p = 0.2039; red, Amoxicillin + SYN-007 (3 mg), p = 0.1688; green, Amoxicillin + SYN-007 (1 mg), p = 0.1796. A similar Shannon alpha diversity analysis including Dog 8 is included as Supplemental Figure 2.

Alpha diversity, using the Shannon Index, was determined for each pre and post treatment sample (Figure 2). Shannon indexes were significantly lower following amoxicillin alone exposure, compared to pretreatment (p = 0.0063). In contrast, no significant differences in calculated Shannon indices were observed pre and post treatment for amoxicillin + SYN-007 at any dose (p = 0.2039, 0.1688, and 0.1796 for SYN-007 10 mg, 3 mg, and 1 mg, respectively). A pretreatment sample from the SYN-007 10 mg cohort (Dog 8) was concluded to be an outlier (Supplemental Figure 1) and omitted from the Shannon alpha diversity analysis displayed in Figure 2. For comparison, this sample was included in the Shannon alpha diversity analysis displayed as Supplemental Figure 2. Importantly, even with the inclusion of Dog 8, Shannon alpha diversity of the SYN-007 10 mg cohort was not significantly different pre and post treatment (p = 0.0632; Supplemental Figure 2).

**Table 1.** Bacterial species richness of fecal microbiomes before and after antibiotic exposure.

Group	Bacterial Species (mean ± SD)		Significance
	Pretreatment	Post-treatment	P value
Amox Alone	298 ± 21	136 ± 30	0.0002
Amox + SYN-007 (10 mg)	294 ± 33	174 ± 60	0.0519
Amox + SYN-007 (3 mg)	278 ± 14	180 ± 49	0.0870
Amox + SYN-007 (1 mg)	260 ± 25	171 ± 20	0.4295
All	283 ± 27	-	-

Notes: Data are displayed as mean ± SD. P values were obtained using the Kruskal-Walllis non-parametric ANOVA with Dunn's Multiple Comparisons Test (GraphPad Prism 7). Comparison of All pretreatment to each pretreatment group were p = 0.7260, >0.9999, 0.9219, and 0.3468 for Amoxicillin Alone, Amox + SYN-007 (10 mg), Amox + SYN-007 (3 mg), and Amox + SYN-007 (1 mg), respectively.

**Figure 3.**
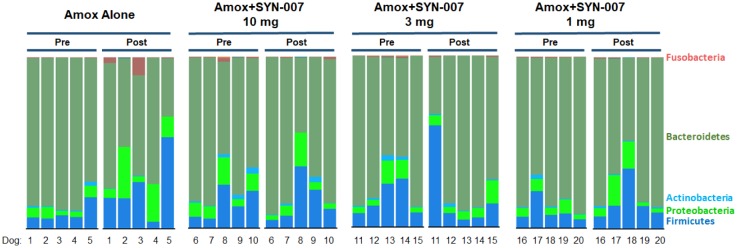
Phyla level stacked bar graph of fecal microbiomes. Notes: Fecal microbiomes for each animal at each time point were analyzed via stacked bar graph comparing pretreatment to post-treatment. The predominant phyla are identified on the right, animal numbers are displayed on the bottom, and treatment groups and collection time point displayed at the top.

**Figure 4.**
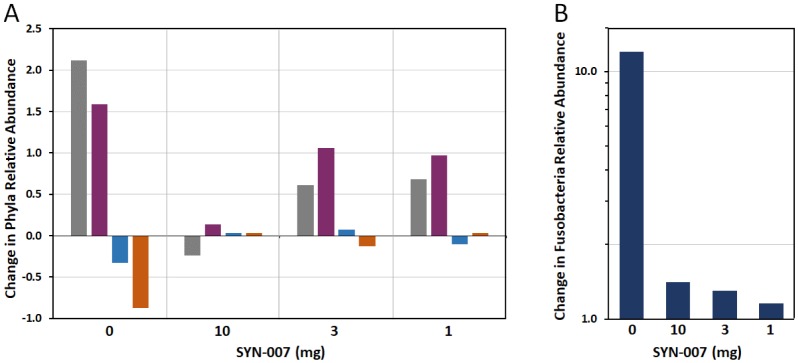
Changes in relative abundance of phyla. Notes: A. Change in relative abundance (mean) of indicated phyla for each cohort from pretreatment to post-treatment. A negative value indicates reduction in relative abundance, positive value indicates increased relative abundance, and zero value represents no change in relative abundance. Gray: Proteobacteria, violet: Firmicutes, blue: Bacteroidetes, orange: Actinobacteria. B. Change in relative abundance (mean) of bacteria in the Fusobacteria phyla (log scale). Positive values indicate increased relative abundance and a value of 1.0 indicates no change in relative abundance.

Specific changes in microbiota composition of the canine microbiomes were observed using stacked bar graphs of bacterial phyla, based on relative abundance in each sample (Figure 3). For ease of comparison, changes in the relative abundance of each phyla after amoxicillin +/− SYN-007 exposure for each cohort are displayed as a bar graph (Figure 4). Amoxicillin alone resulted in the most dramatic alterations of microbiome composition compared to pre treatment microbiomes, including large increases in Firmicutes and Proteobacteria phyla abundance, and decreases in Bacteroidetes and Actinobacteria phyla (Figure 4A). For all amoxicillin + SYN-007 cohorts, changes in phyla abundance were attenuated compared to amoxicillin alone. Notably, SYN-007 10 mg microbiomes displayed the least fluctuation in phyla relative abundance, with slight decreases in Proteobacteria and increases in Firmicutes. SYN-007 3 mg and 1 mg cohorts both displayed increases in Proteobacteria and Firmicutes, with relative abundance of Bacteroidetes and Actinobacteria remaining relatively unchanged. (Figure 4A). In addition, a greater than 10-fold increase in relative abundance of the Fusobacteria phylum, specifically due to *Fusobacterium mortiferium*, was observed in the amoxicillin alone cohort, while this phylum displayed minimal increases in all SYN-007 cohorts (Figure 4B). Notably, one animal from the SYN-007 10 mg cohort, Dog 8, determined to be an outlier (Supplemental Figure 1), displayed the highest pretreatment relative abundance of *F. mortiferium* (2.1%) and bacteria in the Proteobacteria phylum (16.2%), compared to all other pretreatment microbiomes.

Taken together, these gut microbiome data demonstrate that SYN-007 attenuated changes to the gut microbiome caused by oral amoxicillin at all doses evaluated.

## 4. Discussion

Drug dosing optimization is an important and many times overlooked factor for enhancing the sustainability of healthcare [14]. The use of low SYN-007 doses to maintain gut microbiome integrity is expected to improve therapeutic outcomes for patients receiving oral beta-lactam antibiotics while potentially lowering drug costs. Here, a SYN-007 dose response study demonstrated that doses of 10 mg, 3 mg, and 1 mg protected the gut microbiome from damage caused by oral amoxicillin. The highest SYN-007 dose, 10 mg, shown previously to be efficacious in dogs [7],[9], served as the starting point for dose de-escalation. These data demonstrate that SYN-007 doses could be reduced by an order of magnitude while retaining therapeutic utility.

Low SYN-007 doses may also improve efficacy by diminishing the potential for beta-lactamase interference with oral antibiotic systemic absorption. Indeed, following sixteen 1 mg doses of SYN-007, amoxicillin serum concentration PK curves were nearly identical with those of amoxicillin alone including the later time points when amoxicillin serum levels were waning (Figure 1C). This is in contrast to the behavior of the PK curves with higher SYN-007 doses, where amoxicillin serum levels appeared to decrease more rapidly at time points after two hours (Figure 1B). The diminution of systemic amoxicillin concentrations at the 3, 4, and 6 hour time points was apparent with the 3 mg dose, most evident with the 10 mg dose, and reported previously for the 10 mg dose [7],[9]. However, the influence on overall systemic antibiotic levels was minimal as the calculated AUCs of all amoxicillin + SYN-007 curves were not significantly different from that of amoxicillin alone (Figure 1). A likely explanation is that trace amounts of the beta-lactamase were released prematurely in the upper small intestine with the free enzyme degrading a small fraction of the orally-delivered amoxicillin prior to its complete systemic absorption. With administration of low SYN-007 doses, beta-lactamase leakage was reduced to levels that did not cause detectable antibiotic degradation.

Similar to the observations reported here using the low (1 mg) SYN-007 dose, following delivery of 10 mg SYN-007 doses with oral amoxicillin/clavulanate, an antibiotic and beta-lactamase inhibitor combination drug, amoxicillin serum PK curves were indistinguishable with and without SYN-007 [9]. These data suggest that any beta-lactamase enzyme present in the small intestine was neutralized by the beta-lactamase inhibitor and prevented antibiotic degradation [9]. Therefore, the beta-lactamase inhibitor functioned as a fail-safe mechanism to counteract inadvertent enzyme release from SYN-007 in the upper small intestine [9]. The use of lower enzyme doses is an alternative yet complementary strategy to the administration of beta-lactamase inhibitors with both approaches functioning to offset the potential for interference with oral beta-lactam antibiotic systemic absorption.

Here, we demonstrated that SYN-007 protected the gut microbiome from damage caused by oral amoxicillin at all SYN-007 doses tested. SYN-007 preserved both bacterial richness and alpha diversity, and mitigated microbiota composition changes compared to amoxicillin administration without SYN-007. However, pretreatment microbiome composition was surprisingly variable in this dog population, much more so than in previous studies [7],[9]. As stress and diet changes are known to affect gut microbiome composition [15],[16], animals were acclimated for several weeks prior to collection of a baseline fecal sample with the intention of stabilizing gut microbiomes. Therefore, a limitation to this study was the use of small cohorts of five animals each. However, despite the microbiome variability, some patterns did emerge.

Exposure to amoxicillin alone caused a marked increase in abundance of bacteria belonging to the Proteobacteria, Firmicutes, and Fusobacteria phyla, which was reduced in the presence of SYN-007 (Figure 4A). The highest (10 mg) SYN-007 dose appeared most effective in mitigating changes to Proteobacteria and Firmicutes phyla, however, both the 3 mg and 1 mg doses lessened antibiotic-mediated phyla alterations. These data are consistent with a previous study demonstrating an increase in bacterial species within Proteobacteria and Firmicutes phyla resulting in a shift of microbial balance toward Gram-negative bacteria following oral amoxicillin administration in dogs [17]. Changes within the Fusobacteria phyla was exclusively due to one species, *F. mortiferum*, present at low levels in all pretreatment animals and increased in abundance more than 10-fold in amoxicillin alone treated dogs (Figure 4B). With SYN-007, *F. mortiferum* abundance increased less than 50%. *F. mortiferum*, an anaerobic Gram-negative bacteria that is a normal inhabitant of the oropharyngeal and GI tracts, is associated with serious soft-tissue infections in humans and animals [18]. The significance of *F. mortiferum* overgrowth remains unclear and requires additional investigation.

## 5. Conclusions

Optimization of drug dosing is an important consideration for improving healthcare sustainability [14]. Here, a dose response study of SYN-007, a delayed-release formulation of the beta-lactamase, ribaxamase, was performed using doses of 10 mg, 3 mg and 1 mg co-delivered with oral amoxicillin. SYN-007, at all doses evaluated, mitigated antibiotic-mediated gut microbiome alterations demonstrating that that SYN-007 doses could be reduced by an order of magnitude while retaining therapeutic utility. The use of low SYN-007 doses to maintain gut microbiome integrity is expected to improve therapeutic outcomes for patients receiving oral beta-lactam antibiotics while potentially lowering drug costs.
