# Supplementary material for: Low dose oral beta-lactamase protects the gut microbiome from oral beta-lactam-mediated damage in dogs
Source: AIMS Public Health. 2019 Nov 12;6(4):477–87. doi: 10.3934/publichealth.2019.4.477 (PMC6940571; doi:10.3934/publichealth.2019.4.477)
Supplement: Supplementary file 1 [file publichealth-06-04-477-s001.pdf]

AIMS Public Health, 6(4): 477–487.
DOI: 10.3934/publichealth.2019.4.477
Received: 05 September 2019
Accepted: 05 November 2019
Published: 12 November 2019

AIMS Public Health

http://www.aimspress.com/journal/aimsph

Research article

Low dose oral beta-lactamase protects the gut microbiome from oral beta-lactam-mediated damage in dogs

Sheila Connelly[1,]*, Brian Fanelli[2], Nur A. Hasan[2], Rita R. Colwell[2,3] and Michael Kaleko[1]

[1] Synthetic Biologics, Inc., Rockville, MD, United States
[2] CosmosID, Inc., Rockville, MD, United States
[3] University of Maryland Institute for Advanced Computer Studies, College Park, MD, United States

* **Correspondence:** Email: sconnelly@syntheticbioloigics.com; Tel: +2402383866.

Supplementary

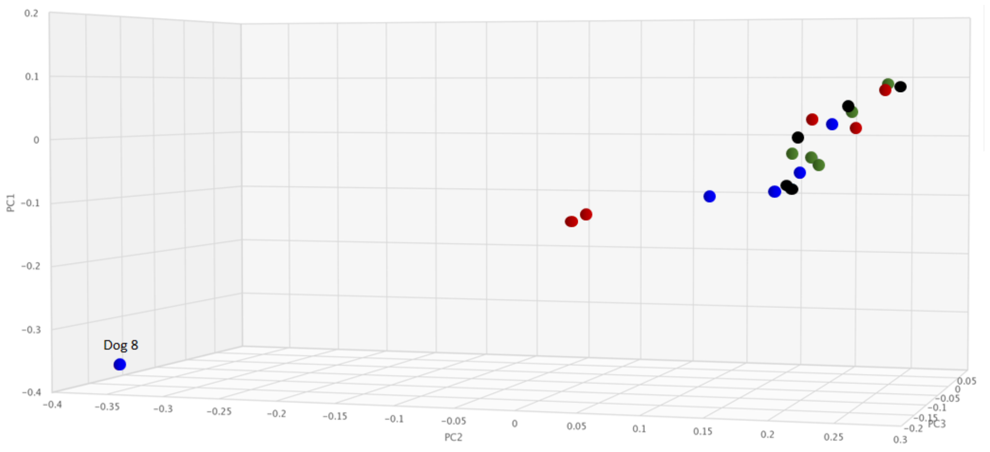

Supplemental Figure 1. Principal coordinate analyses of pretreatment fecal microbiomes. Notes: Pretreatment fecal microbiome for each animal was subjected to principal coordinate analysis using Bray-Curtis dissimilarity [19]. Black: Amoxicillin alone; blue: Amoxicillin + SYN-007 (10 mg); red: Amoxicillin + SYN-007 (3 mg); green: Amoxicillin + SYN-007 (1 mg). Dog 8 (Amoxicillin + SYN-007 (10 mg)) pretreatment microbiome sample was considered an outlier based on this analysis.

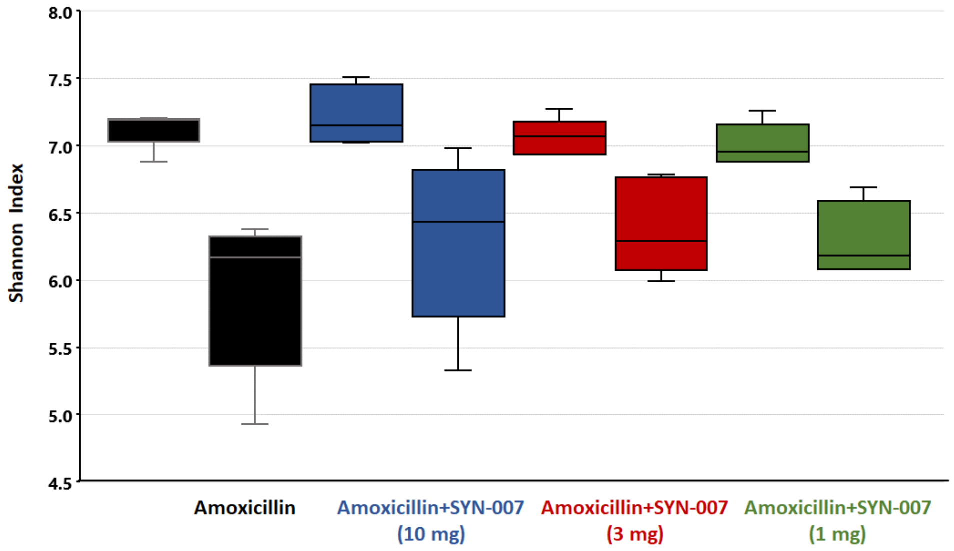

Supplemental Figure 2. Comparison of dog fecal microbiome Shannon alpha diversity prior to and after antibiotic treatment including Dog 8 pre and post treatment samples. Notes: Fecal microbiome metagenomics data were analyzed by Shannon index and are displayed for each cohort as box plots (n = 5). P values were obtained by comparing pretreatment Shannon indexes (Pre) to post-treatment Shannon indexes (Post) of each cohort using Kruskal-Wallis non-parametric ANOVA with Dunn's Multiple Comparisons test (Graphpad Prism 7). Black, Amoxicillin alone, p = 0.0067; blue, Amoxicillin + SYN-007 (10 mg) with Dog 8, p = 0.0632; red, Amoxicillin + SYN-007 (3 mg), p = 0.1766; green, Amoxicillin + SYN-007 (1 mg), p = 0.1951. Compare to Figure 2.

AIMS Press

© 2019 the Author(s), licensee AIMS Press. This is an open access article distributed under the terms of the Creative Commons Attribution License (http://creativecommons.org/licenses/by/4.0)
